# The Relationship between Maternal Plasma Leptin and Adiponectin Concentrations and Newborn Adiposity

**DOI:** 10.3390/nu9030182

**Published:** 2017-02-23

**Authors:** Natália P. Castro, Verônica V. Euclydes, Fernanda A. Simões, Lourdes R. A. Vaz-de-Lima, Cyro A. De Brito, Liania A. Luzia, Delan Devakumar, Patrícia H. C. Rondó

**Affiliations:** 1 Nutrition Department, School of Public Health, University of São Paulo, São Paulo 01246-904, Brazil; natalia@usp.br (N.P.C.); agapito.fe@gmail.com (F.A.S.); lianialuzia@usp.br (L.A.L.); 2 Postgraduate Program in Applied Human Nutrition (PRONUT), University of São Paulo, São Paulo 05508-000, Brazil; veronicaeuclydes@hotmail.com; 3 Immunology Center, Adolfo Lutz Institute, São Paulo 05403-000, Brazil; lourlima@uol.com (L.R.A.V.-d.-L.); cbrito@usp.br (C.A.D.B.); 4 Institute for Global Health, University College London, London WC1N 1EH, UK; d.devakumar@ucl.ac.uk

**Keywords:** leptin, adiponectin, adiposity, mother, newborn

## Abstract

Increased maternal blood concentrations of leptin and decreased adiponectin levels, which are common disturbances in obesity, may be involved in offspring adiposity by programming fetal adipose tissue development. The aim of this study was to assess the relationship between maternal leptin and adiponectin concentrations and newborn adiposity. This was a cross-sectional study involving 210 healthy mother-newborn pairs from a public maternity hospital in São Paulo, Brazil. Maternal blood samples were collected after delivery and leptin and adiponectin concentrations were measured by enzyme-linked immunosorbent assay. Newborn body composition was estimated by air displacement plethysmography. The association between maternal leptin and adiponectin concentrations and newborn adiposity (fat mass percentage, FM%) was evaluated by multiple linear regression, controlling for maternal age, socioeconomic status, parity, pre-pregnancy body mass index (BMI), weight gain, gestational age, and newborn age at the time of measurement. No relationship was found between maternal leptin and FM% of male or female newborn infants. Maternal adiponectin (*p* = 0.001) and pre-pregnancy BMI (*p* < 0.001; adj. *R*^2^ = 0.19) were positively associated with FM% of newborn males, indicating that maternal adiponectin is involved in fetal fat deposition in a sex-specific manner. Large-scale epidemiological, longitudinal studies are necessary to confirm our results.

## 1. Introduction

Obesity is a global health problem and a risk factor for a number of chronic diseases [1,2]. The rise in obesity rates among women of childbearing age is particularly worrisome as studies report an association between obesity during pregnancy and obesity [3] and other adverse health outcomes in adult offspring [4,5]. It is well recognized that early life events have a long-term influence on disease risk later in life, a phenomenon known as “early life programming” in which the fetus makes adaptations to an adverse intrauterine environment to increase its immediate chance of survival [6]. These later-life changes become maladaptive, increasing the risk for a range of chronic diseases such as obesity [3], diabetes, and hypertension [6,7]. Cnattingius et al. [3] suggested a generational cycle of obesity as mothers who were born large for gestational age have a higher risk of obesity and are more likely to have a large-for-gestational-age baby, indicating that obesity may be programmed in utero.

In addition to increased fat mass, obesity has been associated with altered secretion and concentrations of adipokines. Leptin, a hormone produced mainly by adipose tissue, is recognized for its role in the central control of appetite but has also been implicated in platelet aggregation and arterial thrombosis, contributing to obesity-associated cardiovascular disease risk [8,9]. Adiponectin, a cytokine also produced by adipose tissue, is known for its insulin sensitization action and anti-atherogenic properties [8,10]. Maternal blood leptin concentration has been previously linked to newborn fat mass percentage [11]. Similarly, adiponectin has also been associated with offspring adiposity. In an animal model [12], adiponectin gene–knockout dams were fed a high-fat diet for six weeks and were mated to wild-type (WT) sires. Analysis of fetal weight and body composition showed that the maternal high-fat diet increased the weight and fat mass of offspring of WT dams, while the offspring of knockout dams had a lower weight and fat mass. In a recent study [13], the offspring of adiponectin gene–knockout dams exhibited a greater weight and fat mass than WT offspring. Furthermore, chronic infusion of adiponectin in pregnant mice has been shown to decrease fetal growth, possibly by reducing amino acid transporter activity and expression [14]. These adipokines may play important roles in fetal development and possibly induce permanent metabolic changes by programming fat tissue development.

Normal pregnancy is characterized by a series of physiological adaptations, including hormonal changes that alter maternal metabolism and allow fetal growth and nutrition. Some of the metabolic changes that occur during pregnancy resemble those observed in obesity. Weight gain, hyperglycemia, insulin resistance, and altered leptin and adiponectin concentrations are common effects observed in both pregnancy and obesity. Maternal leptin levels increase during pregnancy [15,16], possibly as a consequence of placental production and fat mass gain. Adiponectin levels decrease throughout pregnancy [17,18] and are inversely related to adiposity gain.

In view of the worldwide increase in obesity among pregnant women and the contribution of maternal obesity to offspring obesity, the aim of this study was to investigate the association between maternal plasma concentrations of leptin and adiponectin and newborn adiposity.

## 2. Experimental Section

### 2.1. Experimental Design and Subjects

In this cross-sectional study, 210 women were randomly selected between March and July 2013 from a large maternity hospital in São Paulo city, Brazil, based on their hospital records and antenatal cards. The parameters adopted for sample size calculation were a power of the test of 90% and significance of 0.05 to detect differences in newborn adiposity between normal weight and obese mothers according to Catalano et al. [19]. Exclusion criteria were: adolescence, multiple pregnancies, diabetes, hypertension, hormonal disorders, chronic infectious diseases, drug, tobacco and/or alcohol consumption, preterm (<37 weeks) and post-term (≥42 weeks) delivery, low birth weight (<2500 g), and genetic disorders in the newborn.

Shortly after delivery, the women were invited to participate in the study. A questionnaire was applied to evaluate demographic, socioeconomic (education and housing conditions: television sets, CD players, vehicles, washing machines, DVD players, refrigerators/freezers, bathrooms), and obstetric variables. Mothers were asked to recall their weight prior to pregnancy for calculation of pre-pregnancy body mass index (BMI) and the information was confirmed with the antenatal cards. The following data were collected from the hospital records: maternal date of birth, maternal weight in all antenatal visits, date and time of birth of the infant, type of delivery, sex, gestational age, birth weight, length, head and chest circumferences at birth, and Apgar score. Gestational age was determined by a combination of ultrasonography performed up to the 20th week of gestation, the Capurro method [20] determined between 12 and 48 h after birth, and the last menstrual period. When there was a one-week discrepancy between at least two of the gestational age determinations, one of them was chosen, giving preference to the order of the methods cited above.

### 2.2. Biochemical Assays

Maternal blood was collected within 24–72 h after delivery after an overnight fast into ethylenediaminetetraacetic acid (EDTA)-treated plain vacutainer tubes (Becton Dickinson, Rutherford, NJ, USA) and was immediately centrifuged at 1600× *g* for 10 min. Plasma samples were stored at −20 °C for leptin and adiponectin analysis. These adipokines were determined by enzyme-linked immunosorbent assay (ELISA) (Raybio^®^, Norcross, GA, USA). All biochemical assays were performed in duplicate. The detection limit for leptin was 17.33 pg/mL, with an intra-assay coefficient of variation (CV) of 7.8% and inter-assay CV of 14.52%. The detection limit for adiponectin was 192 pg/mL, with intra- and inter-assay CVs of 4.2% and 8.7%, respectively.

### 2.3. Maternal Anthropometry

Maternal weight and height were determined with a bioelectrical impedance analyzer (Inbody370, Biospace^®^, Seoul, Korea) and a Tonelli stadiometer (model 120A, Tonelli, Criciúma, SC, Brazil), respectively, according to Lohman et al. [21]. These measurements were made 24–72 h after delivery. Pre-pregnancy BMI was calculated using the self-reported pre-pregnancy weight and the measured height. Weight gain during pregnancy was defined as the difference between the maternal weight measured within one week prior to delivery and the maternal weight recorded in the first antenatal visit.

### 2.4. Newborn Anthropometry and Body Composition Assessment

Newborn length was measured with a Seca^®^ infantometer (model 416, Seca, Hamburg, Germany) to the nearest 0.1 cm. Weight was measured to the nearest 1 g using the scale of the air displacement plethysmograph (PEA POD^®^, Cosmed, San Francisco, CA, USA). The Anthro software (version 3.2.2, WHO, Geneva, Switzerland) was used to determine weight-for-age and length-for-age *z*-scores. Body composition was assessed by air displacement plethysmography (PEA POD^®^). All measurements were performed within 24–72 h after birth.

### 2.5. Statistical Analysis

The relationship of newborn fat mass percentage (FM%, outcome or dependent variable) with maternal leptin and adiponectin concentrations (exposure or independent variables) and other independent variables of interest (age, socioeconomic status, parity, pre-pregnancy BMI, weight gain during pregnancy, gestational age, and newborn age at the time of measurement) was determined by univariate analysis according to sex. Variables of biological importance with *p* ≤ 0.20 in the univariate analysis were selected for entry into two multiple linear regression models stratified by sex. A stepwise backward selection method was used for linear regression analysis, considering a *p*-value ≤ 0.05 to be significant. Statistical analysis was performed using the Stata software (version 11; Stata Corp., College Station, TX, USA).

### 2.6. Ethical Considerations

This study was conducted according to the guidelines of the Declaration of Helsinki and all procedures involving human subjects were approved by the Ethics Committees of the School of Public Health, University of São Paulo, Escola Dr. Mário de Moraes Altenfelder Silva Maternity, and Adolfo Lutz Institute (Approval Number 00749812.5.3001.0059).

## 3. Results

Table 1 shows the baseline characteristics of the subjects enrolled in the study. The mean maternal age (SD) was 25.87 (5.18) years. Most mothers considered themselves to be mulatto (43.3%) and had a monthly household income of less than three Brazilian minimum wages (one Brazilian minimum wage = R$678.00 or US$317.00). Fourteen mothers refused to report their income. According to pre-pregnancy BMI, 9.7% of the women were underweight, 28.7% were overweight, and 11.1% were obese. Most women did not gain weight during pregnancy according to the Institute of Medicine (IOM) recommendations [22]. More than half the newborn infants were girls (54.3%) and 76.2% had a birth weight between 3000 and 4000 g. As can be seen in Table 1, 78.1% and 70.5% of the infants had adequate weight-for-age and length-for-age measurements, respectively [23].

In univariate regression analysis, adiponectin, pre-pregnancy BMI and weight gain had a *p*-value ≤ 0.20 and were included in the multiple linear regression model for newborn males. Since pre-pregnancy BMI and weight gain are frequently used together [18], the weight gain variable was maintained in the final model because of its biological significance. For females, none of the maternal or newborn variables had *p* ≤ 0.20. Therefore, only maternal leptin and adiponectin concentrations were selected for the model.

Multivariate regression analysis showed an interaction between sex and adiponectin, but not between sex and pre-pregnancy BMI or adiponectin and pre-pregnancy BMI. In addition, no interaction was observed between sex, adiponectin and pre-pregnancy BMI. Multiple linear regression analysis with newborn FM% as the outcome is shown in Table 2. The FM% of male newborn infants was positively associated with maternal adiponectin concentration (*p* = 0.001) and maternal pre-pregnancy BMI (*p* < 0.001) (adjusted *R*^2^ = 0.19).

## 4. Discussion

In this study, maternal leptin concentration was not associated with fetal adiposity. This result differs from the study of Josefson et al. [11] in which maternal leptin concentration was positively associated with newborn FM%. The difference between the findings of Josefson et al. and our results might be explained by the timing of the blood sampling. In the present study, maternal blood was collected 24 to 72 h post-partum, while in the study of Josefson et al. [11] blood samples were obtained between 36–38 weeks of pregnancy. Since the metabolism of pregnant women is constantly changing, the timing of blood collection is crucial. Leptin is produced by the placenta [24] and maternal plasma concentrations during pregnancy reflect adipose tissue and placental production of this adipokine. Maternal leptin concentration should therefore be measured during the post-partum period rather than during pregnancy when it is difficult to distinguish between the amount of hormone produced by the placenta and that produced by maternal adipose tissue [25]. Furthermore, a cohort study showed that the leptin concentration tends to increase throughout pregnancy. Although obese women begin pregnancy with higher leptin concentrations, they show a lower increase in leptin than their normal weight counterparts as pregnancy progresses [16], in part due to their lower weight gain. Maternal leptin concentrations are proportional to pre-pregnancy BMI and weight gain. In late pregnancy, obese women have only 1.2 times higher circulating leptin levels than normal weight women [16].

In a cohort study, Misra et al. [15] found no association between the magnitude of variation in maternal leptin concentration and variation in infant birth weight, a surrogate measure of FM%. However, an increase in the rate of change in maternal serum leptin in the second half of pregnancy was associated with a reduction in birth weight in overweight/obese pregnant women. While this finding may be related to changes in placental leptin production, it does not reflect the influence of maternal adipose tissue on fetal development. Placental size is known to be positively associated with birth weight [26], and probably also with leptin production [27]. Although hyperleptinemia has been implicated in placental dysfunction in previous studies [28], this is still controversial [29] and we found no evidence of an association between maternal leptin and newborn FM%.

The maternal plasma concentration of adiponectin was positively associated with the FM% of male but not of female newborn infants. Sexual dimorphism is known to occur during fetal development [30,31]. According to our results, differences may exist in the mechanisms involved in newborn male and female fat mass development. To our knowledge, there are no studies in the literature that investigated the association between maternal adiponectin and newborn adiposity by plethysmography, and birth weight is frequently used as a proxy for newborn adiposity. In a recent study, Vernini et al. [32] observed a positive association between maternal adiponectin and birth weight, in agreement with our results. However, the results of studies on this topic are conflicting. While some authors have found an inverse relationship between maternal adiponectin and newborn adiposity [33,34], others report no association between maternal adiponectin and perinatal outcomes [35]. Lowe et al. [34] observed an inverse relationship between newborn adiposity (estimated by skinfolds) and maternal adiponectin concentration between 24 and 32 weeks of gestation. Methodological differences could explain the different results. The study of Lowe et al. [34] was a multicenter study with a heterogeneous population and included women with pre-eclampsia, a condition known to affect the adiponectin concentration [36]. The population of our study was healthy and homogeneous, was selected from the same maternity unit, and had a similar socioeconomic status. In addition, the method used for body composition estimation, air displacement plethysmography, is more accurate [37] than the skinfold measurements used by Lowe et al. [34]. 

Some mechanisms can be suggested to explain how maternal adiponectin may increase FM% of the newborn. Adiponectin plays a role in glucose utilization and fatty acid oxidation by activating AMP-protein kinase, an enzyme involved in many metabolic processes [38]. Adiponectin has also been implicated in the muscle translocation of glucose transporters, particularly glucose transporter type 4 (GLUT4), which would contribute to glucose uptake [39]. It is possible that adiponectin controls glucose uptake by the placenta via activation of AMP-protein kinase and translocation of glucose transporters. In this respect, higher concentrations of adiponectin may increase glucose uptake by the placenta and contribute to fetal fat deposition. However, further studies concerning the placenta are needed to elucidate how maternal adiponectin influences fetal fat deposition. Although animal studies have shown how maternal adiponectin could affect fetal fat metabolism [12,40,41], our study was the first to present that maternal adiponectin concentrations may participate in fetal fat development in a sex-specific manner.

In this study, boys were potentially more vulnerable to the maternal adiponectin concentration than girls, although further studies are necessary to investigate a possible causal relationship. It is known that boys are born heavier than girls, while girls have a greater FM% than boys. In a rabbit model investigating the effect of a maternal high-fat/cholesterol diet [42], males were more affected by the high-fat/cholesterol diet than females. The authors concluded that placental adaptations to the diet differed between sexes. Bellisario et al. [43] reported a sex-dependent resiliency to stressful and metabolic challenges following prenatal exposure to a high-fat diet, i.e., female offspring were more resistant to the maternal high-fat diet than male mice. In humans, O’Tierney-Ginn et al. [44] reported an association between maternal pre-pregnancy BMI and fat mass and fat-free mass of newborn males, expanding the current knowledge of sex differences in fetal body composition. However, the issue is still controversial, considering that in the study of Au et al. [45] newborn female sex, maternal Caucasian ethnicity and increased gestational weight gain were the variables most strongly associated with increased FM%.

The main limitation of this study was the lack of collection of adipose tissue samples from women undergoing a cesarean section for the determination of adipokine expression. Haghiac et al. [17] evaluated patterns of adiponectin expression in term pregnancy and observed decreased adiponectin expression in obese women when compared to normal weight women. Thus, in addition to plasma concentrations, it would be interesting to investigate the association between maternal leptin and adiponectin expression and newborn adiposity. Moreover, a larger sample size would permit a three-way interaction analysis between sex, maternal adiponectin and pre-pregnancy BMI.

## 5. Conclusions

The maternal leptin concentration was not associated with fetal adiposity, while adiponectin was positively associated with adiposity in male newborn infants. Longitudinal studies are needed to confirm the existence of a sex-specific relationship between maternal adiponectin and fetal fat development.

## Figures and Tables

**Table 1:** Demographic, socioeconomic, obstetrics, clinical and biochemical characteristics of the study population (*n* = 210).

**Maternal Characteristics**			
	*n*	%	Mean (SD)
Age (years)			25.87 (5.18)
Ethnicity			
White	76	36.2	
Mulatto	91	43.3	
Black	32	15.2	
Native American	8	3.9	
Unknown	3	1.4	
Household income (BMW in R$)			1770.52 (1200.17)
<1	17	8.1	
1–1.9	65	30.9	
2–2.9	72	34.3	
3–4.9	27	12.9	
≥5	15	7.1	
Unknown	14	6.7	
Parity			
1	76	36.2	
>1	134	63.8	
Height (m)			1.60 (0.06)
<1.50	13	6.2	
1.50–.60	89	42.4	
1.6–1.69	97	46.2	
≥1.70	11	5.2	
Pre-pregnancy BMI (kg/m^2^)			24.60 (5.24)
Underweight (<18.5)	19	9.7	
Normal weight (18.5–24.9)	98	50.3	
Overweight (25.0–29.9)	56	28.7	
Obese (≥30.0)	22	11.1	
Weight gain during pregnancy (kg)			12.97 (5.76)
Below IOM recommendations	49	25.4	
Within IOM recommendations	67	34.7	
Above IOM recommendations	77	39.9	
Type of birth			
Vaginal			
Normal	99	47.1	
Forceps	51	24.3	
Cesarean section	60	28.6	
Leptin (ng/mL)			33.78 (30.21)
0.5–12.2	53	25.2	
12.3–24.3	53	25.2	
24.4–44.7	52	24.8	
44.8–173.38	52	24.8	
Adiponectin (µg/mL)			22.58 (16.62)
1.2–11.6	52	24.8	
11.7–17.5	53	25.2	
17.6–27.3	53	25.2	
27.4–86.7	52	24.8	
**Newborn Characteristics and Anthropometry**			
Gestational age (weeks)			39.42 (1.04)
Sex			
Female	114	54.3	
Male	96	45.7	
Birth weight (g)			3377.01 (407.62)
2500–3000	34	16.2	
3000–3500	109	51.9	
3500–4000	51	24.3	
4000–5000	16	7.6	
Weight-for-age (*z*-score)			3197.79 (0.40)
<−2	1	0.5	
−2–−1	29	13.8	
−1–1	164	78.1	
1–2	14	6.7	
≥2	2	0.9	
Length-for-age (*z*-score)			49.30 (1.81)
<−2	5	2.4	
−2–−1	36	17.1	
−1–1	148	70.5	
1–2	20	9.5	
≥2	1	0.5	
Fat mass percentage			8.93 (4.18)
Female	114	54.3	9.91 (4.20)
Male	96	45.7	7.76 (3.86)

BMW: Brazilian minimum wage = R$678.00 per month (1 US$ = 2.14 R$).

**Table 2:** Multiple linear regression analysis considering the fat mass percentage of male (*n* = 91) and female (*n* = 107) newborn infants as outcomes.

**Male FM%**	**Coefficient**	**95% CI**		***p***
Leptin (ng/mL)	−0.023	−0.054	0.008	0.143
Adiponectin (µg/mL)	0.076	0.031	0.121	0.001
Pre-pregnancy BMI (kg/m^2^)	0.332	0.153	0.512	0.000
Weight gain during pregnancy (kg)	−0.012	−0.108	0.084	0.804
*R*^2^ = 0.22; adjusted *R*^2^ = 0.19				
**Female FM%**	**Coefficient**	**95% CI**		***p***
Leptin (ng/mL)	−0.008	−0.035	0.017	0.517
Adiponectin (µg/mL)	0.015	−0.033	0.063	0.543
*R*^2^ = 0.006; adjusted *R*^2^ = −0.012				

FM%: fat mass percentage; 95% CI: 95% confidence interval.
